# Serum Inflammatory Biomarkers Contribute to the Prognosis Prediction in High-Grade Glioma

**DOI:** 10.3389/fonc.2021.754920

**Published:** 2022-01-27

**Authors:** Xiao-Yong Chen, Ding-Long Pan, Jia-Heng Xu, Yue Chen, Wei-Feng Xu, Jin-Yuan Chen, Zan-Yi Wu, Yuan-Xiang Lin, Hong-Hai You, Chen-Yu Ding, De-Zhi Kang

**Affiliations:** ^1^ Department of Neurosurgery, Neurosurgical Research Institute, The First Affiliated Hospital, Fujian Medical University, Fuzhou, China; ^2^ Department of Radiation Oncology, The Second Affiliated Hospital of Fujian Medical University, Quanzhou, China; ^3^ Department of Medical Oncology, Affiliated Cancer Hospital of Zhengzhou University, Zhengzhou, China; ^4^ Department of Ophthalmology, The First Affiliated Hospital, Fujian Medical University, Fuzhou, China; ^5^ Fujian Key Laboratory of Precision Medicine for Cancer, the First Affiliated Hospital, Fujian Medical University, Fuzhou, China; ^6^ Key Laboratory of Radiation Biology of Fujian higher education institutions, the First Affiliated Hospital, Fujian Medical University, Fuzhou, China

**Keywords:** serum inflammatory biomarker, LASSO, nomogram, prognosis, glioma, SVM

## Abstract

**Background:**

To evaluate the prognostic value of serum inflammatory biomarkers and develop a risk stratification model for high-grade glioma (HGG) patients based on clinical, laboratory, radiological, and pathological factors.

**Materials and Methods:**

A retrospective study of 199 patients with HGG was conducted. Patients were divided into a training cohort (n = 120) and a validation cohort (n = 79). The effects of potential associated factors on the overall survival (OS) time were investigated and the benefits of serum inflammatory biomarkers in improving predictive performance was assessed. Univariable and multivariable Cox regression analyses, the least absolute shrinkage and selection operator (LASSO) regression analysis, and support vector machines (SVM) were used to select variables for the final nomogram model.

**Results:**

After multivariable Cox, LASSO, and SVM analysis, in addition to 3 other clinico-pathologic factors, platelet-to-lymphocyte ratio (PLR) >144.4 (hazard ratio [HR], 2.05; 95% confidence interval [CI], 1.25–3.38; *P* = 0.005) were left for constructing the predictive model. The model with PLR exhibited a better predictive performance than that without them in both cohorts. The nomogram based on the model showed an excellent ability of discrimination in the entire cohort (C-index, 0.747; 95%CI, 0.706–0.788). The calibration curves showed good consistency between the predicted and observed survival probability.

**Conclusion:**

Our study confirmed the prognostic value of serum inflammatory biomarkers including PLR and established a comprehensive scoring system for the OS prediction in HGG patients.

## Introduction

High-grade glioma (HGG), such as World Health Organization (WHO) grade III and IV, is the most common type of intracranial malignant tumor. The inherent high heterogeneity of HGG contributes to poor therapeutic efficacy as the dominant factor, resulting to the high mortality rates and rapid progression. The median survival time of grade IV patients is only 15 months (1–3). In addition, in clinical practice, the overall survival (OS) for HGG patients who receive the same treatment may differ significantly at the individual level (4). Therefore, summarizing the different characteristics and identifying effective prognostic factors based on retrospective reviews could stratify the patients for personalized follow-up regimen development and further individualized management improvement of prognosis. Clinically, adequate and reliable prognosis prediction for HGG patients is urgently need but remains challenging.

Inflammation plays a crucial role in tumor microenvironment and tumor progression, namely, glioma. Therefore, inflammatory biomarkers, such as neutrophil-to-lymphocyte ratio (NLR), platelet-to-lymphocyte ratio (PLR), lymphocyte–monocyte ratio (LMR), lactate dehydrogenase (LDH), may not only reflect inflammation status but also indicate glioma progression. Currently, many factors including serum inflammatory factors, have been identified as effective prognostic factors in HGG (5–8). However, the predictive value of serum inflammatory biomarkers in glioma prognosis remains controversial (9). Therefore, the role of serum inflammatory biomarkers needs to be further investigated due to the controversial results. In addition, considering the high heterogeneity of HGG, a single risk factor may be limited in precisely and effectively predicting prognosis. A multivariable model by comprehensively integrating the clinical, laboratory, and pathological risk factors may be more effective and reliable for prognosis prediction.

Hence, our study aims to investigate the predictive value of serum inflammatory biomarkers in HGG, and further develop and validate a risk stratification model for HGG based on risk factors extracted from clinical, laboratory, radiological, and pathological information.

## Materials and Method

### Study Population

The medical records of 199 patients diagnosed as HGG who underwent surgery for tumor resection at the First Affiliated Hospital of Fujian Medical University between January 2015 and January 2020 were retrospectively reviewed. Patients were divided into two cohorts: the training cohort (n = 120) and the validation cohort (n = 79). It was approved by the ethics committee of the First Affiliated Hospital of Fujian Medical University and conformed to the ethical guidelines of the Declaration of Helsinki (ethical number: MRCTA, ECFAH of FMU [2020]005). The requirement of informed consent was waived due to its retrospective design.

The eligibility criteria for inclusion were: (1) Diagnosis of HGG was confirmed by pathological examination; (2) full data of preoperative routine blood test (i.e., serum LDH level, neutrophil, monocyte, and lymphocyte counts) were available; (3) no history of surgical treatment, chemotherapy, or radiotherapy before admission; (4) no hematological system disorder, other neurological diseases, impaired liver function, or other systemic diseases.

### Data Collection

The clinical information, namely, age, sex, preoperative Karnofsky performance status (KPS), comorbid condition, treatment regimens (concurrent chemoradiotherapy, CCRT), and preoperative serum routine tests, namely, white blood cell, neutrophil, monocyte, lymphocyte, and platelet counts and LDH level were extracted from medical records. Based on preoperative magnetic resonance imaging (MRI), features of tumor consist of tumor size, location, and peritumoral edema diameter were also included in our analysis. Parameters in the MRI were independently evaluated by two neuroradiologists who were blind to the patient information. In addition, pathological and immunohistochemical information including grade (III or IV), isocitrate dehydrogenase (IDH) mutation, and Ki-67 index (<10% or ≥10%) were collected for analysis. In addition to LDH, other serum inflammatory markers including NLR, PLR, and LMR were defined as follows. NLR = neutrophil/lymphocyte, PLR = platelet/lymphocyte, LMR = lymphocyte/monocyte. OS time was defined as the interval from operation to death. Patients were censored in those who did not die at the end of follow-up.

Using the receiver operating characteristic (ROC) curve analysis based on the OS rate, the cut-off values of the several serum inflammatory biomarkers were determined: NLR = 2.31, PLR = 144.4, LMR = 4.47, and LDH = 171 U/L. Patients were subsequently divided into two groups based on the cut-off values.

### Build-Up of Models and Establishment of Nomogram

To determine the value of serum inflammatory biomarkers in prognosis prediction, two models were produced and compared for the selection of final model. ModelA consisted of all independent risk factors without serum inflammatory biomarkers, while ModelB consisted of all independent risk factors such as serum inflammatory biomarkers. After comparing the predictive performance and clinical utility in both training and validation cohort, the better model was selected as the final model to establish a nomogram in the entire cohort.

### Statistical Analysis

Continuous variables were presented as mean ± standard deviation for 2-sample *t-*test as they fitted normal distribution. The other continuous variables were presented as median (range) and analyzed by non-parametric test. Categorical data were described as frequency (percentage) and compared by Pearson χ^2^ test or Fisher exact test. The optimal cut-off values of NLR, PLR, LMR, and LDH for OS prediction were determined by ROC curve analysis.

The univariable and multivariable Cox proportional hazards regression models were applied to evaluate the prognostic significance of variables. Those variables with *P <*0.10 in univariable analysis were further analyzed by multivariable analysis. After that, the least absolute shrinkage and selection operator (LASSO) regression analysis and support vector machines (SVM) was used to select the possible variables for the model. Time-dependent ROC curve was applied to assess the accuracy and effectiveness of predictive models at different time points. Decision curve analyses (DCA), Integrated Discrimination Improvements (IDI), and Net Reclassification Index (NRI) were performed to evaluate and compare the clinical usage of different models. After comparing the performance of different models, the final model based on all the possible prognostic factors was used to construct a nomogram to predict the probability of survival at 1, 2, and 3 years. The performance of the nomogram was evaluated by Harrell’s concordance index (C-index), DCA, ROC, and the calibration curves.

All statistical analyses were performed using SPSS 17.0 statistical software (SPSS, Inc., Chicago, Illinois, USA) and R statistical software (R version 4.0.3, R Project, www.r-project.org). All statistical tests were two-sided and *P <*0.05 was considered as statistically significant.

## Results

### Patient Characteristics

The baseline clinical, laboratory, and pathological characteristics of the two cohorts are presented in **Table 1**. The proportion of death (*P* = 0.146) showed gratifying similarity between the two cohorts. The median OS time of training cohort and validation cohort was similar, 14.50 (10.00–23.00) months vs 14.00 (8.00–19.00) months, *P* = 0.293. In addition, the clinical parameters, laboratory data, tumor features, surgical factors, and pathological parameters showed no significant difference between the two cohorts. Overall, the selected parameters in the two cohorts showed high homogeneity and comparability, revealing that the collection of data were reliable with high quality.

**Table 1:** Characteristics of patients in the training and validation cohorts.

Characteristic	All (n = 199)	Training cohort (n = 120)	Validation cohort (n = 79)	*P*-value
**Demographics**				
Age, year				0.569
<60	143 (71.9%)	88 (73.3%)	55 (69.8%)	
≥60	56 (28.1%)	32 (26.7%)	24 (30.4%)	
Sex				0.150
Male	111 (55.8%)	62 (51.7%)	49 (62.0%)	
Female	88 (44.2%)	589 (48.3%)	30 (38.0%)	
**Functional status**				
KPS score	80 (70–90)	80 (70–80)	80 (70–90)	0.838
**Comorbid condition**				
Hypertension				0.211
No	169 (84.9%)	105 (87.5%)	64 (81.0%)	
Yes	30 (15.1%)	15 (12.5%)	15 (19.0%)	
Diabetes mellitus				0.454
No	190 (95.5%)	113 (94.2%)	77 (97.5%)	
Yes	9 (4.5%)	7 (5.8%)	2 (2.5%)	
**Laboratory data**				
RBC count 10^9^/L	4.61 (4.33–4.91)	4.62 (4.32–4.89)	4.61 (4.34–4.92)	0.922
HCT	0.41 (0.38–0.44)	0.41 (0.38–0.44)	0.41 (0.38–0.45)	0.903
WBC count 10^9^/L	7.18 (5.76–9.55)	7.32 (5.79–10.05)	6.94 (5.76–9.13)	0.155
NEU count 10^9^/L	4.81 (3.30–7.42)	4.89 (3.43–8.14)	4.35 (3.05–6.14)	0.112
MON count 10^9^/L	0.37 (0.29–0.50)	0.37 (0.29–0.48)	0.39 (0.30–0.53)	0.251
LYM count 10^9^/L	1.65 (1.32–2.12)	1.63 (1.30–2.14)	1.66 (1.34–2.02)	0.574
PLT count 10^9^/L	227.00 (196.00–272.00)	226.50 (196.00–269.25)	232.00 (195.00–279.00)	0.787
NLR	2.53 (1.77–5.28)	2.70 (1.83–6.27)	2.33 (1.69–4.26)	0.153
PLR	138.10 (104.58–181.11)	136.71 (109.74–190.25)	140.76 (101.24–175.21)	0.514
LMR	4.53 (3.12–6.00)	4.53 (3.23–6.07)	4.56 (3.10–5.90)	0.786
HB g/L	141.00 (130.00–149.00)	141.50 (132.00–148.75)	140.00 (129.00–149.00)	0.446
HDL mmol/L	1.24 (1.03–1.42)	1.25 (1.07–1.45)	1.21 (0.96–1.40)	0.404
ALB g/L	42.47 ± 3.74	42.71 ± 4.00	42.10 ± 3.30	0.260
LDH U/L	171.00 (150.00–201.00)	174.00 (152.00–204.75)	165.00 (148.00–192.00)	0.132
**Tumor features and surgical factors**				
Location				0.191
Supratentorial	97 (48.7%)	63 (52.5%)	34 (43.0%)	
Infratentorial	102 (51.3%)	57 (47.5%)	45 (57.0%)	
Tumor diameter, cm	4.92 ± 1.66	4.97 ± 1.75	4.84 ± 1.51	0.596
Peritumor edema cm	2.26 (1.30–3.09)	2.20 (1.13–3.00)	2.40 (1.81–3.10)	0.094
Tumor crossing midline				0.256
No	140 (70.4%)	88 (73.3%)	52 (65.8%)	
Yes	59 (29.6%)	32 (26.7%)	27 (34.2%)	
Extent of resection				0.409
GTR	157 (78.9%)	97 (80.8%)	60 (75.9%)	
STR	42 (21.1%)	23 (19.2%)	19 (24.1%)	
**Pathological grade and Immunohistochemistry**				
WHO grade				0.998
III	83 (31.7%)	38 (31.7%)	25 (31.6%)	
IV	136 (68.3%)	82 (68.3%)	54 (68.4%)	
IDH mutant				0.119
No	155 (77.9%)	89 (74.2%)	66 (83.5%)	
Yes	44 (22.1%)	31 (25.8%)	13 (16.5%)	
Ki-67				0.126
<10%	41 (20.6%)	29 (24.2%)	12 (15.2%)	
≥10%	158 (79.4%)	91 (75.8%)	67 (84.8%)	
**CCRT**				0.908
No	42 (21.1%)	25 (20.8%)	17 (21.5%)	
Yes	157 (78.9%)	95 (79.2%)	62 (78.5%)	
**Status**				0.146
Alive	70 (35.2%)	47 (39.2%)	23 (29.1%)	
Dead	129 (64.8%)	73 (60.8%)	56 (70.9%)	
**OS month**	14.00 (9.00–21.00)	14.50 (10.00–23.00)	14.00 (8.00–19.00)	0.293

Values are reported as number, number (%), median (25–75%), and mean ± SD.

KPS, Karnofsky performance status; RBC, red blood cell; HCT, hematocrit; WBC, white blood cell; NEU, neutrophil; MON, monocyte; LYM, lymphocyte; PLT, platelet; NLR, neutrophil-to-lymphocyte ratio; PLR, platelet-to-lymphocyte ratio; LMR, lymphocyte–monocyte ratio; HB, hemoglobin; HDL, high density lipoprotein; ALB, albumin; LDH, lactate dehydrogenase; GTR, gross-total resection; STR, subtotal resection; WHO, World Health Organization; IDH, isocitrate dehydrogenase; CCRT, concurrent chemoradiotherapy; OS, overall survival.

### Prognostic Factors of OS in the Training Cohort

The ROC curve analysis showed that NLR = 2.31, PLR = 144.4, LMR = 4.47, and LDH = 171 U/L were the optimal cut-off values (**Table 2**). Based on the optimal cut-off values, the area under curve (AUC) of NLR, PLR, LMR, and LDH were 0.637 (95% confidence interval [CI], 0.544–0.723), 0.624 (95%CI, 0.531–0.711), 0.616 (95%CI, 0.523–0.703), and 0.601 (95%CI, 0.508–0.690), respectively; the sensitivity of NLR, PLR, LMR, and LDH were 69.86, 54.79, 56.16, and 61.64%, respectively, and the specificity of NLR, PLR, LMR, and LDH were 59.57, 74.47, 65.86, and 59.57%, respectively.

**Table 2:** The cut-off value and area under the curve of the serum inflammatory biomarkers.

Parameter	Cut-off value	AUC	Sensitivity (%)	Specificity (%)	95%CI of AUC
NLR	2.31	0.637	69.86	59.57	0.544–0.723
PLR	144.4	0.624	54.79	74.47	0.531–0.711
LMR	4.47	0.616	56.16	65.86	0.523–0.703
LDH	171	0.601	61.64	59.57	0.508–0.690

AUC, area under curve; CI, confidence interval; NLR, neutrophil-to-lymphocyte ratio; PLR, platelet-to-lymphocyte ratio; LMR, lymphocyte–monocyte ratio; LDH, lactate dehydrogenase.

The univariable analysis showed that Age ≥60 years (HR, 2.12; 95%CI, 1.27–3.53; *P* = 0.007), NLR >2.31(HR, 2.14; 95%CI, 1.29–3.53; *P* = 0.003), PLR >144.4 (HR, 2.51; 95%CI, 1.56–4.03; *P <*0.001), LMR ≤4.47 (HR, 1.79; 95%CI, 1.12–2.84; *P* = 0.014), LDH >171 U/L (HR, 2.20; 95%CI, 1.37–3.55; *P* = 0.001), tumor crossing midline (HR, 1.60; 95%CI, 0.98–2.60; *P* = 0.061), WHO IV grade (HR, 5.31; 95%CI, 2.69–10.47; *P <*0.001), and Ki-67 ≥10% (HR, 3.88; 95%CI, 1.97–7.63; *P <*0.001) were associated with decreased OS time (**Table 3**). In contrast, high KPS score (HR, 0.96; 95%CI, 0.94–0.98; *P <*0.001), IDH mutant (HR, 0.29; 95%CI, 0.15–0.56; *P <*0.001), and CCRT (HR, 0.42; 95%CI, 0.25–0.70; *P* = 0.001) were significantly associated with increased OS time. In multivariable analysis, PLR >144.4 (HR, 2.05; 95%CI, 1.25–3.38; *P* = 0.005), LDH >171 U/L (HR, 1.82; 95%CI, 1.11–2.99; *P* = 0.017), WHO IV grade (HR, 6.20; 95%CI, 2.93–13.13; *P <*0.001), and Ki-67 ≥10% (HR, 3.08; 95%CI, 1.52–6.23; *P <*0.001) were independently associated with decreased OS time (**Table 3**). On the contrary, high KPS score (HR, 0.96; 95%CI, 0.93–0.98; *P* = 0.001), IDH mutant (HR, 0.46; 95%CI, 0.23–0.91; *P* = 0.026), and CCRT (HR, 0.29; 95%CI, 0.16–0.52; *P <*0.001) were independently associated with improved OS time.

**Table 3:** Univariable and multivariable analysis of OS in the training cohort.

Parameter	Univariable analysis			Multivariable analysis		
	HR	95%CI	*P*-value	HR	95%CI	*P*-value
Age ≥60 years	2.12	1.27–3.53	0.007			
High KPS score	0.96	0.94–0.98	<0.001	0.96	0.93–0.98	0.001
NLR >2.31	2.14	1.29–3.53	0.003			
PLR >144.4	2.51	1.56–4.03	<0.001	2.05	1.25–3.38	0.005
LMR ≤4.47	1.79	1.12–2.84	0.014			
LDH >171 U/L	2.20	1.37–3.55	0.001	1.82	1.11–2.99	0.017
Tumor crossing midline	1.60	0.98–2.60	0.061			
WHO grade						
III		Reference			Reference	
IV	5.31	2.69–10.47	<0.001	6.20	2.93–13.13	<0.001
IDH mutant	0.29	0.15–0.56	<0.001	0.46	0.23–0.91	0.026
Ki-67 ≥10%	3.88	1.97–7.63	<0.001	3.08	1.52–6.23	0.002
CCRT	0.42	0.25–0.70	0.001	0.29	0.16–0.52	<0.001

OS, overall survival; HR, hazard ratio; CI, confidence interval; KPS, Karnofsky performance status; NLR, neutrophil-to-lymphocyte ratio; PLR, platelet-to-lymphocyte ratio; LMR, lymphocyte–monocyte ratio; LDH, lactate dehydrogenase; WHO, World Health Organization; IDH, isocitrate dehydrogenase; CCRT, concurrent chemoradiotherapy.

### Comparison Between Models With or Without Inflammatory Biomarkers in the Training and Validation Cohorts

**Figure 1:**
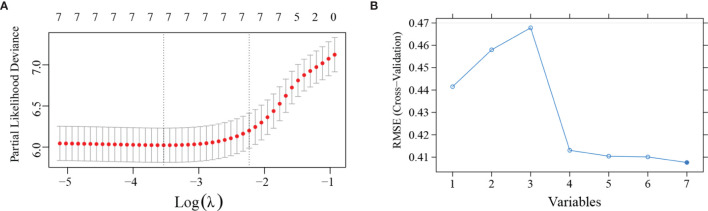
Least absolute shrinkage and selection operator (LASSO) regression analysis and support vector machines (SVM) was applied to further identify prognostic factors in the training cohort. **(A)** LASSO regression analysis showed that the 7 variables were all left in the LASSO model based on the partial likelihood deviance vs log (λ). The right dotted vertical line was drawn at the optimal value of λ by one standard error of the minimum criteria. **(B)** SVM showed that the model consists of the top 4 variables almost reached the lowest value of Root Mean Square Error (RMSE) based on 10-fold cross-validation. The blue curve represents the different value of RMSE based on models consists of different variables. Lower values of RMSE represents better consistency between prediction and actuality.

The Lasso regression model and SVM was used together to further identify prognostic factors for the OS (**Figures 1A, B**). In the Lasso regression analysis, the optimal λ value of 0.11 was selected (one standard error of the minimum criteria) and resulted in 7 non-zero coefficients (**Figure 1A**). In the SVM, the model consists of top 4 variables (rankings: WHO grade, KI-67 index, IDH mutation, and PLR) almost reach the lowest value of Root Mean Square Error (0.4131) based on 10-fold cross-validation. Thus, combined with the results of Lasso regression analysis and SVM, 4 variables were left after screening. Based on the 4 independent prognostic factors, we established two models: ModelA, consisting of WHO grade, KI-67 index, IDH mutation; and ModelB, consisting of WHO grade, KI-67 index, IDH mutation, and PLR.

**Figure 2:**
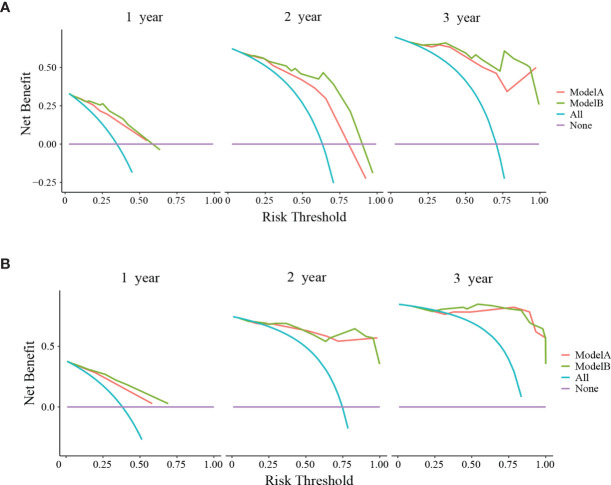
Decision curve analyses (DCA) of ModelA and ModelB at 1, 2, and 3 years after surgery in the training cohort **(A)** and 1, 2, and 3 years after surgery in the validation cohort **(B)**. The y-axis represents the net benefit and the x-axis represents the corresponding risk threshold. The blue line represents that all patients die during the follow-up. The purple line represents that no patients die during the follow-up. In the most points of risk threshold, ModelB (green line) showed more benefits in predicting survival status than ModelA (red line).

**Figure 3:**
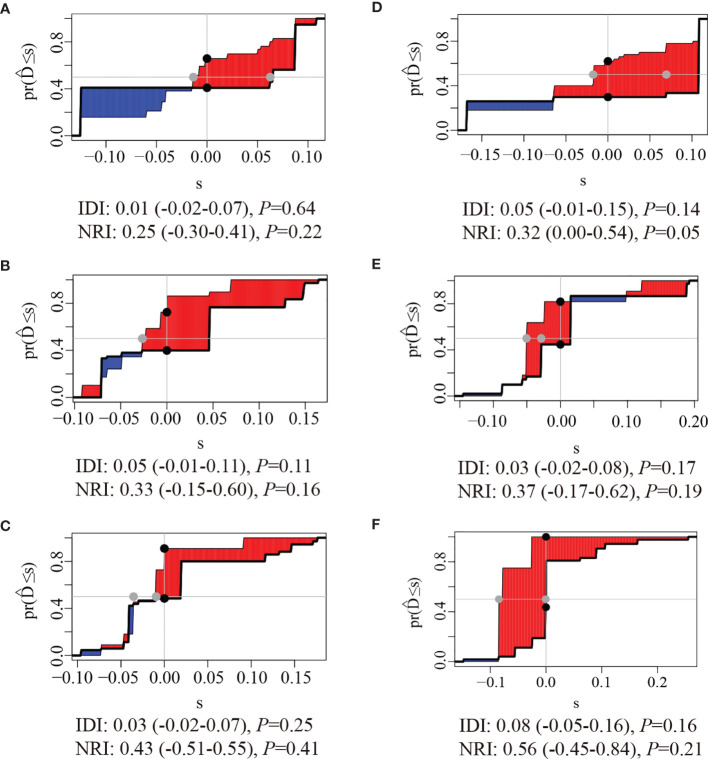
Integrated Discrimination Improvements (IDI) and Net Reclassification Index (NRI) of ModelB comparing to ModelA at **(A)** 1 year, **(B)** 2 years, and **(C)** 3 years after surgery in the training cohort and **(D)** 1 year **(E)**, 2 years, and **(F)** 3 years after surgery in the validation cohort. the red areas were greater than blue areas and the median value of NRI and IDI were all greater than zero, indicating that the predictive ability of ModelB may be better than ModelA.

**Figure 4:**
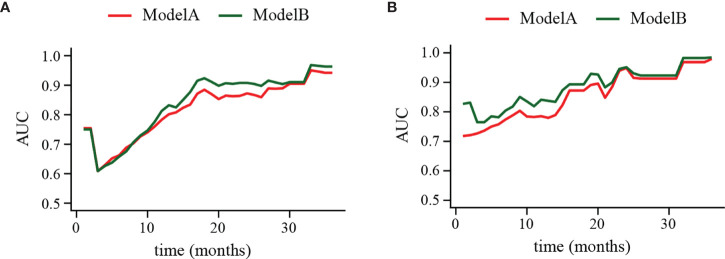
Time-dependent receiver operating characteristic (ROC) curve of ModelA and ModelB in the training **(A)** and validation cohort **(B)**. The y-axis represents the area under curve (AUC) and the x-axis represents the follow-up time. In the most points of follow-up time, the AUC value of ModelB (green line) was higher than ModelA (red line).

The clinical usage was evaluated by DCA, IDI, and NRI: the 1-, 2-, and 3-year DCA curves in the training cohort and validation cohort for the two prediction models showed that the maximum net benefit of ModelB was better than ModelA (**Figure 2**); as shown in **Figure 3**, the IDI approach indicated that the clinical utility of ModelB may be better than ModelA in both training cohort (1 year after surgery: IDI = 0.01, 95%CI = −0.02–0.07, *P* = 0.64; 2 years after surgery: IDI = 0.05, 95%CI = −0.01–0.11, *P* = 0.11; 3 years after surgery: IDI = 0.03, 95%CI = −0.02–0.07, *P* = 0.25) and validation cohort (1 year after surgery: IDI = 0.05, 95%CI = −0.01–0.15, *P* = 0.14; 2 years after surgery: IDI = 0.03, 95%CI = −0.02–0.08, *P* = 0.17; 3 years after surgery: IDI = 0.08, 95%CI = −0.05−0.16, *P* = 0.16); the NRI approach shown in **Figure 3** indicated that the clinical utility of ModelB may be better than ModelA in both training cohort (1 year after surgery: NRI = 0.25, 95%CI = −0.30−0.41, *P* = 0.22; 2 years after surgery: NRI = 0.33, 95%CI = −0.15–0.60, *P* = 0.16; 3 years after surgery: NRI = 0.43, 95%CI = −0.51–0.55, *P* = 0.41) and validation cohort (1 year after surgery: NRI = 0.32, 95%CI = 0.00–0.54, *P* = 0.05; 2 years after surgery: NRI = 0.37, 95%CI = −0.17–0.62, *P* = 0.19; 3 years after surgery: NRI = 0.56, 95%CI = −0.45–0.84, *P* = 0.21). The time-dependent ROC curves showed that the AUC of both models in the two cohorts were all over 0.7 and ModelB exhibited better performance at most time points (**Figure 4**). The above results showed that ModelB exhibited better predictive performance than ModelA in the both training and validation cohorts and was selected as the final model.

### Establishment and Verification of Nomogram

**Figure 5:**
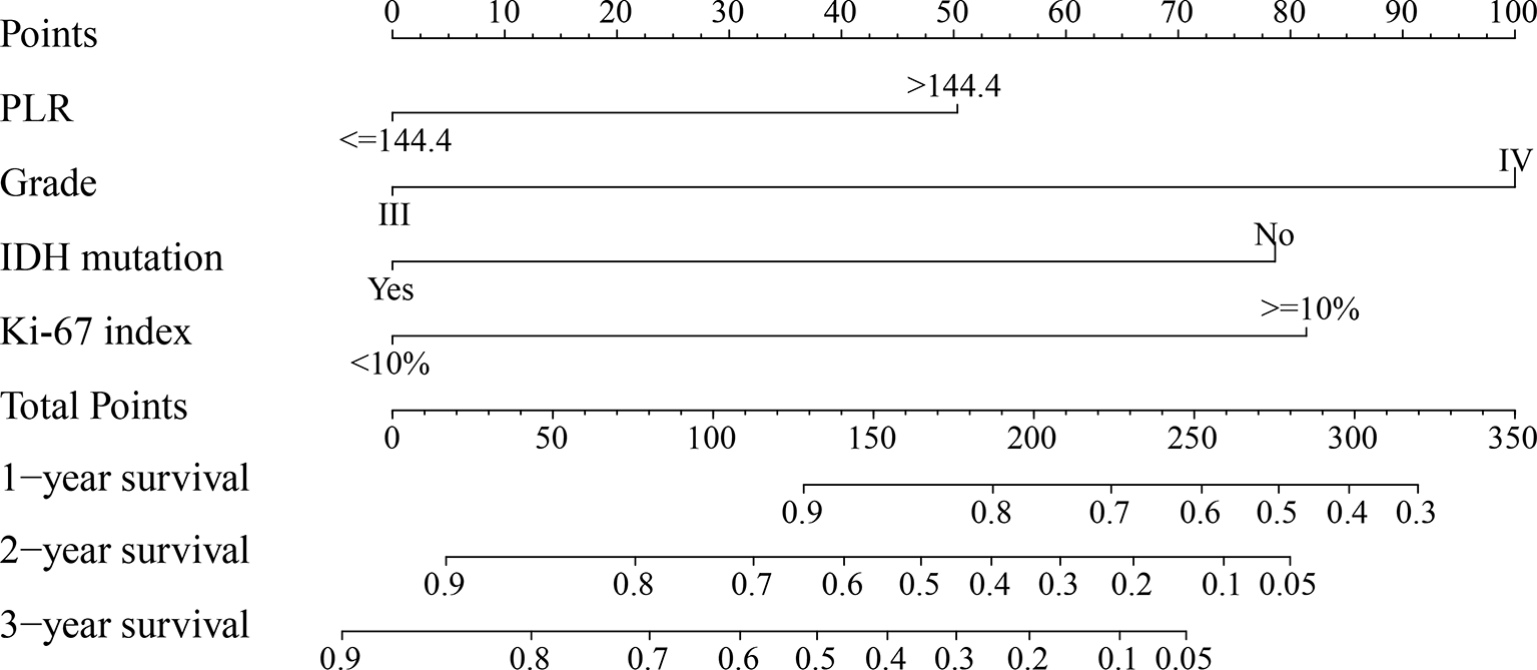
The nomogram for predicting 1-, 2-, and 3-year survival rates of high-grade glioma patients. For each variable, draw a straight line up to the Points axis to calculate the point. After summing the points and locating it on the Total Points axis, draw a straight line down to the 1-year survival, 2-year survival, and 3-year survival axis to determine the probability of surviving for 1, 2, and 3 years. PLR, platelet-to-lymphocyte ratio; IDH, isocitrate dehydrogenase.

**Figure 6:**
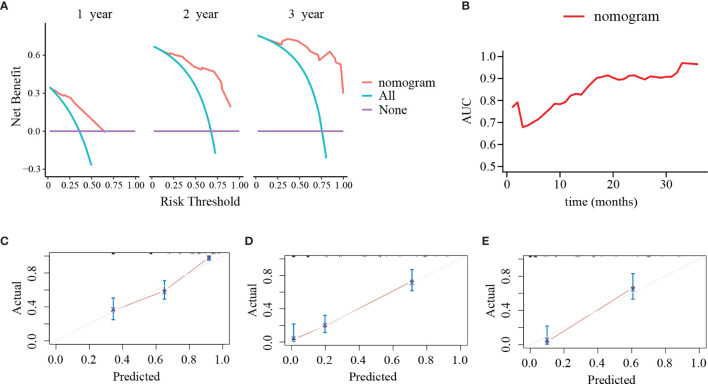
Decision curve analyses (DCA), time-dependent receiver operating characteristic (ROC) curve and calibration curves of the nomogram. **(A)** DCA of the nomogram at 1, 2, and 3 years after surgery. The y-axis represents the net benefit and the x-axis represents the corresponding risk threshold. The blue line represents that all patients die during the follow-up. The purple line represents that no patients die during the follow-up. The red line represents the net benefits of nomogram at different risk threshold. **(B)** The predictive value of the nomogram at different points of follow-up after surgery. **(C–E)** The calibration curves of the nomogram to predict **(C)** 1-year, **(D)** 2-year, and **(E)** 3-year survival rates. The y-axis represents actual survival and the x-axis represents the predicted survival probability based on nomogram. The gray oblique line represents the ideal prediction and the red line represents the performance of the nomogram. Close fit to the grey oblique line indicates the consistency between the predicted and observed survival probability.

PLR level, WHO grade, IDH mutation, and KI-67 index were incorporated into the nomogram for OS prediction in the entire cohort (**Figure 5**). The nomogram showed an excellent ability of discrimination (C-index, 0.747; 95%CI, 0.706–0.788). The DCA curves and time-ROC curves showed that the nomogram had excellent clinical utility and predictive performance (**Figures 6A, B**). The calibration curves for the OS rate at 1-, 2-, and 3-year showed good consistency between the predicted and observed survival probability (**Figures 6C–E**).

## Discussion

Currently, the maximal safe resection combined with concurrent chemoradiotherapy is considered as the most effective treatment regimen for HGG. Even so, patients who underwent the same treatment regimen have exhibited significant difference in their prognosis. With the rapid development of precision medicine, individualized treatment and follow-up strategy were urgently needed. Precise and reliable survival models could contribute to guide clinicians in formulation of treatment plan and management of individual patients. Among different tumor types, nomograms integrating multiple prognostic factors into a reliable tool has been widely applied in predicting survival of patients (10, 11).

Considering the interactions among the related factors may better reflect the complexity of malignant tumor (12). In this study, we attempted to incorporate factors that may have an effect on survival. In the pursuit of simplicity, serum inflammatory biomarkers in our study were handled by a categorical manner as they are actually continuous variables. This may result in a considerable loss of statistical power with introduction of bias in the multiple regression analysis (13). However, considering those inflammatory biomarkers as categorical variables may be more appropriate in guiding process of clinical decision-making as continuous variables could not necessarily increase the prediction performance (14). Therefore, the serum inflammatory biomarkers in our study were divided as binary variables and valued as 0 or 1 based on their optimal cut-off value instead of absolute value.

Our study utilized the Lasso regression analysis and SVM to analyze the independent prognostic risk factors left after multivariable Cox regression analysis. Comparing to the Cox regression analysis, the Lasso regression analysis is a new approach for variables selection by analyzing all variables at the same time. It could minimize the coefficients and produce some coefficients which are exactly zero. Hence, it decreases the estimation variance and provides appropriate prognostic factors with non-zero coefficients (15). In addition, as a widely-used machine learning-based analysis (16), SVM was also utilized in our study to screen variables. Based on the multivariable Cox regression analysis, Lasso regression analysis, and SVM, PLR value, tumor grade, IDH mutation, and Ki-67 index were independent prognostic factors for OS time. In addition, the results showed that GTR was not significant associated with OS in HGG patients, while advanced age lost its significance in the multivariable Cox analysis. This may be caused by the relatively small sample size and high heterogeneity of the disease.

All the above independent factors were considered as potential prognostic indicators of glioma or other malignancies in the previous studies. In malignant tumors, the evoked inflammatory responses and inflammatory cytokines and mediators regulated by tumor microenvironment played an essential role in tumor progression (17–19). PLR served as a biomarker of systemic inflammation and has been considered as an independent prognostic factor in many solid tumors (20). The IDH genotype, an important genetic hallmark of glioma, has been definitely confirmed its prognostic value (21). There is no doubt that the prognosis of glioma is negatively associated with the tumor grade due to its important indication for malignancy of glioma (22). As a proliferation index, Ki-67 has been widely considered as an effective predictor of prognosis and adjuvant therapy responsiveness (23, 24).

Inflammation response could weaken the immune response to malignant tumor (25), permitting serum inflammatory biomarkers serve as prognostic factors. However, whether they are valuable and how to comprehensively utilize these predictors to predict the prognosis of patients with HGG remains unclear. Thus, based on the 4 independent prognostic factors, we established two models. DCA, IDI, and NRI were used to evaluate the clinical utility of the predictive models. Time-dependent ROC compared the predictive performance among the two different models at various time points instead of a fixed time to determine the final model, which was another advantage of our study. Based on time-ROC analysis, DCA, IDI, and NRI, the model including PLR level showed better performance than another model without the two biomarkers, which was selected as the final model to establish a nomogram.

In our study, the discrimination and calibration abilities of the nomogram were evaluated to ensure its accurate application. Herein, we proposed that the training cohort and validation cohort were homogeneous and comparable based on the results of statistical analysis. C-index could reflect the probability of patients with shorter OS time ranked with higher risk of death according to the model in a random selection process. Therefore, the C-index comprehensively considered both the occurrence of the event and the follow-up duration, which is particularly suitable for time-to event analysis. In the entire cohort, the nomogram showed a strong discriminating capability concluded by high C-index value, which were 0.747 (95%CI, 0.706–0.788). The DCA curves and ROC curves showed that the nomogram had excellent clinical utility and predictive performance. In addition, calibration curve is an evaluation method of the agreement between the observed and predicted prognosis in a predictive model. The calibration curves showed good consistency between the predicted and observed survival probability, indicating the ideal repeatability and reliability of the nomogram.

The most important finding of our study is confirmation of the prognostic value of serum inflammatory biomarkers in HGG patients and further construction of a nomogram that could accurately and reliably predict prognosis of HGG patients in a relatively small sample. Functional status, inflammatory condition, adjuvant treatment, pathological grade and immunochemical features were all taken into account when the nomogram for OS prediction was established. By using a simple and intuitive graphical representation, the nomogram enabled its application in clinical care of individual patient. Specifically, total points of nomogram based on the 4 associated factors could be calculated for individual patients. Thus, the corresponding estimated survival probability could be obtained and used to guide clinicians for making individualized follow-up management. Identifying high-risk patients based on precious prediction and taking timely measures may improve their outcome.

The present study also several inherent limitations that should be discussed. First, as a retrospective and single-institution study with a relatively small sample, it may have been subject to interference and selection bias. Second, some clinical, laboratory, and immunohistochemical factors were not included in our study due to the lack of examination or incomplete medical records. Particularly, some molecular profiling may have impacts on glioma prognosis but could not be available due to the retrospective design. Third, the validity and generalizability of the nomogram required to be validated in other independent patient groups. Fourth, the training and validation samples were not in the same time frame which may cause bias due to advancements in medical practice. Final, though the predictive model consisted of adequate prognostic factors is beneficial to HGG management, it should be well aware that the inherent high heterogeneity in HGG is still hard to settle. Further prospective study is needed to minimize the limitations in our study.

## Conclusion

Taken together, our study confirmed the prognostic value of serum inflammatory biomarkers and established a comprehensive scoring system for the OS prediction in HGG patients. The clinical nomogram could assist clinicians when making survival predictions and follow-up management in individual HGG patient. Further studies with larger sample size are required to verify our findings.

## Data Availability Statement

The raw data supporting the conclusions of this article will be made available by the authors, without undue reservation.

## Ethics Statement

The studies involving human participants were reviewed and approved by the local ethics committee of the First Affiliated Hospital of Fujian Medical University. The ethics committee waived the requirement of written informed consent for participation.

## Author Contributions

D-ZK, C-YD, and H-HY performed and designed the study, and wrote the manuscript. X-YC, D-LP, J-HX, YC, W-FX, J-YC, Z-YW, and Y-XL collected, interpreted, and analyzed the data. All authors contributed to the article and approved the submitted version.

## Funding

This study was supported by grants from the Excellent talent project (No. YJCRC-B-KDZ2021), the Startup Fund for Scientific Research of Fujian Medical University (No. 2020QH1035), the project of improving the diagnosis and treatment ability of complicated diseases (No. PT-YNBZW2018), and China Postdoctoral Science Foundation (No.2020M682070).

## Conflict of Interest

The authors declare that the research was conducted in the absence of any commercial or financial relationships that could be construed as a potential conflict of interest.

## Publisher’s Note

All claims expressed in this article are solely those of the authors and do not necessarily represent those of their affiliated organizations, or those of the publisher, the editors and the reviewers. Any product that may be evaluated in this article, or claim that may be made by its manufacturer, is not guaranteed or endorsed by the publisher.
